# Risk of Exclusion in People with Disabilities in Spain: Determinants of Health and Poverty

**DOI:** 10.3390/ijerph15102129

**Published:** 2018-09-27

**Authors:** Angel Belzunegui-Eraso, Inma Pastor-Gosálbez, Xavier Puig-Andreu, Francesc Valls-Fonayet

**Affiliations:** 1 Social Inclusion Chair, Rovira i Virgili University, 43002 Tarragona, Spain; xavier.puig@urv.cat; 2 Medical Anthropology Research Center, Rovira i Virgili University, 43002 Tarragona, Spain; inma.pastor@urv.cat; 3 Faculty of Nursing, Rovira i Virgili University, 43002 Tarragona, Spain; francesc.valls@urv.cat

**Keywords:** disability, poverty, health, social exclusion, social inequality

## Abstract

In this paper, we analyze data from the 2012 *Encuesta de Integración Social y Salud* (Social Integration and Health Survey) of the *Instituto Nacional de Estadística* (Spanish National Institute of Statistics) to obtain profiles created by combining disability, poverty and social exclusion. We hypothesize that the probability that people will experience social exclusion increases if they have a disability, chronic illness or limitation in conducting everyday activities, and that this probability is greater for women than for men. To conduct our analysis, we constructed a social exclusion model based on a series of social determinants that acts as a dependent variable. In this context, social exclusion is understood to go beyond the concept of financial poverty. We performed bivariate analyses, in which we calculated the Odds Ratios (OR) for certain variables considered to be predictors of social exclusion. We also performed a means comparison test and an ANOVA test to observe differences between individuals with recognized disability and those without. Finally, we conducted logistic regression analysis to determine which vulnerability profiles are most likely to experience a situation of social exclusion. We also discuss the limitations of our study, and suggest areas in, which the relationships between health, social exclusion and disability can be further investigated.

## 1. Introduction

Health is one of the dimensions in which social determinants, especially those involving social vulnerability, are most intense. The deprivation that negatively affects the chance to enjoy a long and healthy life and leads to chronic health problems is defined by factors, such as low income, lack of security, dependence and lack of social participation. Certain illnesses can become chronic, due to impoverishment and social isolation [1,2]. People with disabilities and the families of those with disabilities have a greater incidence, intensity and level of multidimensional poverty than others. Their levels of deprivation are also higher [3]. The worst subjective evaluation of health status is associated with social vulnerability, especially poverty [4]. Those with limited financial resources have poorer health conditions and lower rates of social participation [5]. We should also point out that the relationship between disability, poverty and social exclusion is arousing greater interest among those responsible for formulating social policies, since individuals with disabilities are being integrated into national poverty reduction programs [6].

For the above reasons we considered it interesting to introduce disability as another factor that determines the probability of experiencing social exclusion. The combination of disability and health deterioration is interesting to study together with the risks of poverty and exclusion. The aim of this article is to analyze the possible association between health, poverty and social exclusion by comparing populations with and without disability. The data we used were taken from the 2012 Social Integration and Health Survey (EISS-2012) [7] conducted by the Spanish National Institute of Statistics (INE). More specifically, we used data from the survey of individuals conducted with a sample *n* = 16,614, in which 2696 people (18.4%) stated that they experienced some form of disability. We also took our definition of disability from the International Classification of Functioning, Disability and Health (ICF) [8] as a generic term covering impairments/deficiencies, activity limitations and participation restrictions. Disability is therefore understood to be the interaction between experiencing a disease (such as cerebral palsy, Down syndrome or depression), and personal and environmental factors (such as negative attitudes, inaccessible public buildings and transportation, or limited social support). Chronic illness, disability and limitations for functioning may be part of the same interaction resulting in a situation of social exclusion. In this study, we used logistic regression analysis based on the interaction of these factors to explore the probability of social exclusion. Functioning may be considered a global term referring to a person’s bodily functions, activities and participation. Similarly, disability encompasses deficiencies, activity limitations and participation restrictions.

**Figure 1:**
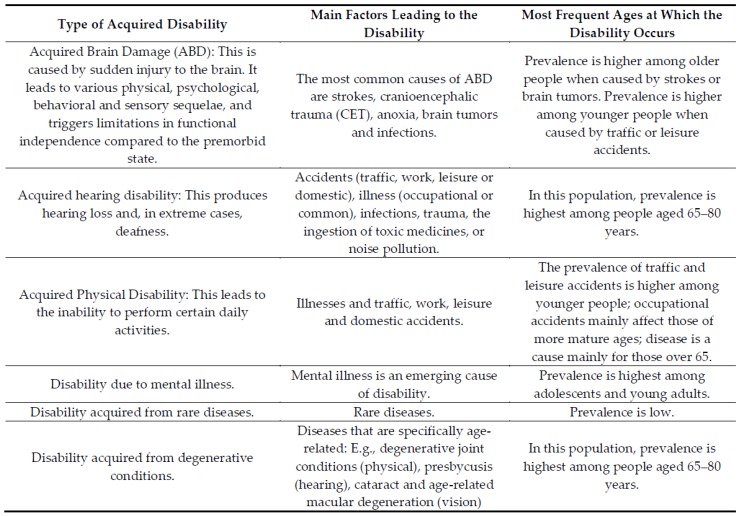
Types of acquired disability. Source: Adapted from Huete et al. (2015) [9].

As we mention below, the EISS-2012 does not differentiate between forms of disability, so we are unable to differentiate between acquired disability and congenital disability. We have therefore used age as a proxy for acquired disability, since most such disabilities occur after 45 years of age. In fact, Huete et al. (2015) [9] showed that two thirds of the population with an acquired disability are concentrated in the older age groups. Congenital disability is therefore more present in the younger age groups. Acquired disability may have various causes, including accidents, due to risk behaviors, which are more frequent among adolescents and young people, and accidents at work, which are more frequent among individuals in what we term the mid- and late productive periods (Figure 1).

We have adopted a multidimensional perspective of poverty, since this provides a broader range of analysis than the financial perspective. The focus of interest is no longer simply an individual or family’s volume of income. Instead, the population’s social conditions are taken into account, and poverty is defined as the result of a set of shortcomings that may be material (income, consumption and possession of other non-financial material goods) or non-material (health, social weakness, marginalization, exclusion, lack of information, lack of training, lack of citizens’ or political rights or lack of exercising those rights, lack of involvement in redistribution mechanisms, fragile social relations, etc.). This approach has been consolidated in the majority of studies and is used by Eurostat to construct social exclusion and poverty indicators.

Similarly, social exclusion is the result of an accumulation of social vulnerability factors inherent to the economic and social system that generate not only inequality, but also the loss of social bonds, disconnection and social marginalization. As well as economic problems, factors, such as health-related issues and, in particular, limitations, due to chronic illnesses and disability, play an important role.

In the first part of this article, we discuss several ideas related to the multidimensionality of the concept of social exclusion, explain the need to separate this concept analytically from that of poverty, and present our hypothesis. We then explain the methodological and analytical aspects of the study. In the results section we describe the differences between the populations with and without disabilities based on several sociodemographic indicators and others that are more specifically related to health. Next, we propose a measure of social exclusion based on a synthetic index of determinants of exclusion from several dimensions that focus on limitations for performing everyday activities. This section includes logistic regression analysis to calculate the probability that an individual with certain characteristics will or will not present social exclusion. We end this paper with a discussion on the scope of our results, the limitations of our study, and the perspectives for new studies on the social exclusion of individuals with disabilities.

## 2. Background

The concept of social exclusion cannot be assimilated with that of financial poverty. Social exclusion comprises various aspects of deprivation and participation, including consumption, political activity and social involvement [10]. The term social exclusion incorporates emerging processes that in the new modernity impede the social integration of many social groups, not only on account of financial inequality [11]. It is a complex process that operates in various dimensions. It implies a lack of resources, goods and services and the inability to participate in leisure and cultural activities, engage in social and political participation or, more broadly speaking, establish social relationships, all of which are activities that most people are able to take part in [12]. Despite a lack of consensus on its definition, the notion of social exclusion is strongly linked to that of social isolation (or the loss of a relationship network) [13].

In our study of people with disabilities, we encountered those who, though not financially impoverished, may be considered excluded, due to certain limitations from activities that most people are able to conduct on a daily basis. In general, the individuals with severe limitations are more likely to be affected by situations of social exclusion, whether or not this includes poverty.

Certain exogenous factors affect the social exclusion processes of people with disabilities. These include architectural barriers and non-accessible urban spaces in general [14], and others related to institutional structures, such as the type of schools, the support of public institutions through social transfers [15], and discrimination in the labor market [16]. Endogenous factors include the type and degree of disability and the chronicity of health problems that may or may not be associated with that disability, as well as aspects, such as a lack of self-confidence.

The lack of a social bond appears increasingly frequently in studies as a factor that most influences social isolation, and, ultimately, social exclusion. For example, the importance of a social network for the elderly and how chronic health problems are intensified in cases of social isolation have been demonstrated [17]. In Spain, studies of this lack of a social bond in women who present problems of marginalization have shown that their state of health deteriorates as their social isolation grows [18]. Other studies have documented how the economic recession in Spain has led to a lack of social bonds in the Spanish population most affected by unemployment and deprived of a relationship environment that for some is their main pattern of sociability [19].

Although health indicators are persistently worse among the population with scarce resources, those at risk of severe social exclusion experience even more acute health-related problems [20]. This occurs in every health dimension (physical, psychological and social) [21].

This is true both for the general population and for those with disabilities [22,23], whose health problems are exacerbated in comparison with the rest of the population. In this regard, a Poverty, Disability and Social Exclusion vicious circle exists that manifests itself when the disabilities occur before retirement and are associated with the living and working conditions of the sectors of society with the lowest levels of income and education, leading to situations of social vulnerability that in extreme cases create poverty and exclusion [24]. Some authors have used Sen’s ability approach to discover the relationship between poverty, disability and gender and shown that women with disabilities are at the greatest risk of poverty. They have also shown that women with disabilities are more disadvantaged than men, especially in developing countries where poverty is combined with traditional negative attitudes towards women. [25,26]. A World Bank study showed that health conditions are a component of disability. When combined with disability, certain chronic diseases and/or poor health in general acquire greater importance in societies with few specific protection systems for the disabled and where universal design is under-developed [27].

In view of the above, we hypothesize that the probability of experiencing social exclusion increases if one is disabled, has a chronic disease, lives in poverty, or is a woman.

## 3. Materials and Methods

### 3.1. Data and Sample

The data analyzed in this study were taken from the 2012 *Encuesta de Integración Social y Salud* (Social Integration and Health Survey) of the *Instituto Nacional de Estadística* (INE, Spanish National Institute of Statistics), and specifically from the module conducted on individuals. This survey is the first conducted by the INE fully adapted to the International Classification of Functioning, ICF 2001, of the World Health Organization (WHO). It has been carried out in all EU countries with a common methodology, and this allows obtaining harmonized information at European level on disability and barriers in the social participation of people with disabilities.

The base sample is very broad (*n* = 16,614), so we divided it into two subsamples, one of which comprised individuals who reported some form of disability (hereafter PWD) (*n*_1_ = 2696), and another, which comprised individuals who did not (hereafter Pw/oD) (*n*_2_ = 13,918).

The questionnaire of the EISS-2012 includes the officially recognized disability through the possession of a document that certifies a degree equal to or greater than 33% of disability. In our sample the 10.1% possess an official certification of disability (a degree equal to or greater than 33%). This number is slightly higher than the percentage of people with disabilities officially recognized in Spain, 8.5% according to the latest INE data.

In addition, those people who without reporting the degree of disability, have severe difficulties or inability to perform certain daily tasks have been added: Eat alone; getting in or out of bed alone; inability to sit up and get up from a chair; dressing and undressing alone; go to the bathroom alone; take a bath or shower alone. We have registered the impossibility of performing these tasks, mainly due to a chronic or long-term illness, and/or mainly due to a limitation in basic activities (difficulties in seeing, hearing, concentrating, moving). The percentage of people with limitations that are additionally collected in our sample is 6.13%. The average age of people with disabilities is 63.5 years. People with certified disability have an average age of 47.5 years and people with limitations added to our sample have an average age of 70.4 years.

In the people with limitations (6.13% of the sample with disability) are predominant degenerative problems derived from age. In officially recognized persons with disabilities (10.1% of the sample with disabilities) there is a greater variety of congenital and acquired disabilities throughout life (before 65 years of age).

A three-stage sampling approach with stratification of the first-stage units was used. The first stage units were the census sections; the second stage units were the main family dwellings, with all households investigated; and the third stage units were the people. The sampling errors for the total number of people with disabilities was 2.21% (3.77% for men and 2.74 for women) [28].

Table 1 shows that the percentages of men and women with disabilities are highest in the over-65 age groups. The average age of people with disability is 63.5 years (the average for men is 61.02 years and the average for women is 64.9 years). The average of people without disability is 47.9 years (the average for men is 47.32 years and the average for women is 48.45 years). Additionally, the average age of people with severe limitation is 65.85 years, while the average age of people without severe limitation is 49.94 years.

### 3.2. Variables

In its 2008 Disabilities, Independence and Dependency Situations Survey, the Spanish National Institute of Statistics divided the variable age into four categories: (1) Primary productive stage (16 to 30 years); (2) middle productive stage (31 to 47 years); (3) late productive stage (48 to 65 years); and (4) retirement (66 years and above). We used these age group to determine whether significant differences exist in relation to the variables studied.

Most of the variables we used were nominal or ordinal. For some analyses we constructed ad hoc indices from the original variables. These indices were treated as ordinal variables and used to simplify the information via additive processes.

The nature of the initial, nominal and ordinal variables ensured that the problem of collinearity, which is more typical of multiple linear regression analysis with metric variables, did not exist. The indices we included had moderate correlations, which overcomes this problem. For prediction analysis, we opted for logistic regression, which enables individuals to be assigned to groups with a dichotomous dependent variable as the destination. The independent variables can be quantitative, ordinal or nominal. Regression coefficients and their significance were assigned to these variables, making it possible to determine which categories (dummy variables) best predict whether the dependent variable belongs to a specific group.

To determine the size of the population living below the poverty line (for a person living alone in Spain in the reference year (2011), this was 8321 euros per year), we started from the variable income by segment in the household of the person surveyed. We combined this variable with the type of household (calculating the thresholds in accordance with the modified scale of the OECD) and the variable *difficulties in meeting certain expenses*. We were thus able to assign the poor/not poor dichotomy to the individuals in the sample (though we should point out that this resulting variable is just an approximation). As no quantitative, direct income indicator exists, we had to choose a proxy variable for poverty. The construct for risk of poverty showed that 22.8% of the sample was living below the poverty line (8321 euros per year for a person living alone) in the year studied. This figure is not substantially different from that reported by other statistical sources, such as the 2012 *Encuesta de Condiciones de Vida* (Living Conditions Survey), which put it at 20.8%. However, as our data slightly overestimates the risk of poverty, this should be taken into account when drawing conclusions. The poor/not poor dichotomous variable was constructed ad hoc from variables of the EISS-2012. As mentioned, it is therefore a proxy variable.

Similarly, we began from a construction of social exclusion (explained more thoroughly in the next section) from various dimensions of the EISS-2012, e.g., social bonds, attendance at cultural shows, Internet use, opportunities for spending time on one’s hobbies, training and employment. The model for constructing a net index of the determinants of social exclusion was an additive one based on the number of determinants of exclusion situations in these dimensions. For comparative purposes, we standardized the net index of social exclusion determinants by creating a Social Exclusion Determinants Index that reflected not so much exclusion itself, but one’s personal situation before the number of determinants that imply that one is excluded from a general set of activities (hobbies, mobility, culture, relationships, employment, training, etc.). We used this index to conduct mean comparison analysis and one-factor ANOVA for those variables that were estimated to potentially be influential factors of social exclusion.

Using the above index, we divided the population into two categories (social exclusion/no social exclusion) based on the average value of the Social Exclusion Determinants Index. The resulting dichotomous variable served as the basis for establishing analyses based on the Odds Ratios (OR).

We should point out that the variable *Limitations for conducting certain activities* divides people into three categories: (a) Those with severe limitation (6.1% of the sample); (b) those with moderate limitation (11.97%); and (c) those with no limitation (81.4%). This classification results from the direct question on the survey: “In at least the last 6 months, to what extent have you felt limited, due to a health problem in performing activities that people usually perform?”.

To address the complexity of social exclusion, we proposed a synthetic index of determinants based on a range of items divided into the following dimensions: Dimension 1—mobility; Dimension 2—accessibility; Dimension 3—education; Dimension 4—employment; Dimension 5—internet access; Dimension 6—contact and social support; and Dimension 7—leisure. Each dimension comprises a series of dichotomous response items weighted from highest to lowest, as follows: (1) Chronic illness or health problems; (2) long-term limitations in performing basic activities; (3) financial reasons (lack of money, i.e., being unable to afford it); (4) lack of technical support that prevents one from leaving the house at will; and (5) lack of assistance that prevents one from leaving the house at will. After calculating the value in each dimension, we analyzed the main components to determine the contribution of each dimension to the new components. We used the correlations of each dimension with the first component of the analysis as the weighting value for each dimension.

Therefore, not all dimensions have the same importance in the later definition of the Determinants of Social Exclusion index. The importance of each dimension is reflected in Table 2.

We then prepared a net Determinants of Social Exclusion index, which is the sum of the weighted values of each dimension. The resulting values for each individual indicate their total number of impediments. The scores were then standardized using the following expression so that their values range between 0 and 1:

$$\mathrm{SID}=\frac{\sum_{D=1}^{n}\left(D_{1}\cdot w_{1}+\ldots +D_{n}\cdot w_{n}\right)}{k}$$

where SID is the Standardized Index of Determinants, *D*_1_ … *D_n_* is the sum of the values of each of the indices representing the dimensions considered, *W*_1_ … *W_n_* are the weights with which each dimension is weighted, and *k* is the maximum value of the determinants in a dimension (in our case, this value is 10). The SID ranges from 0, indicating individuals who have no determinant of social exclusion, to 1, indicating the maximum possible accumulation of social exclusion determinants.

In order to consider people as individuals with the greatest number of determinants of exclusion, we adopted the mean of the distribution while bearing in mind that the distribution is strongly asymmetrical to the right. This type of distribution enables us to identify rare and extreme cases. Provided the interquartile range is small, as it is in this case, the mean can be used as a cut-off value to dichotomize the variable. Individuals were thus grouped into those with a number of determinants above the mean and those with a number of determinants below it. This dichotomous variable served as a construct for calculating individual probabilities of risk of exclusion. To simplify the language, we refer to individuals with or without exclusion. In reality, however, we should bear in mind that what we are really discussing is the number of social determinants that people accumulate that bring them closer to or further away from a situation of exclusion. We should also bear in mind that individuals who share the value 1 (exclusion) for the dichotomous variable do not necessarily have no differences between them. The dichotomous variable tells us whether one has more or fewer than the average number of social determinants, while the net index of determinants of exclusion made it possible to establish a scale of exclusion that we later used to identify those most at risk of social exclusion (i.e., those who accumulate the greatest number of social determinants and, therefore, have the highest score in the net index).

This division shows that 71% of the sample (*n* = 10,343) may be considered not to present social exclusion while 29% (*n* = 4271) may be considered to present exclusion to varying degrees. Note that this is higher than the percentage of people living in poverty, because more dimensions than finance are measured here. This means that an individual who is not poor may score on certain social exclusion items as being isolated or presenting a highly disabling disease that prevents him/her from conducting relational activities. However, trying to identify this high casuistry would be beyond the scope of this article.

### 3.3. Statistical Analyses

All analyses were performed using SPSS version 25 statistical software (IBM, New York, NY, USA). To address the study of association between poverty, health, disability and social exclusion, we first proposed a bivariate analysis in which we differentiated between the population without disability and the population with disability. Bivariate analysis enabled us to observe the differences between the two populations which in most situations presented statistical significance (chi-square tests for *p* = 0.05).

We then performed logistic regression analysis (Logit) to determine the probability of experiencing a situation of social exclusion. At this juncture, we can advance that this probability increases if one has some form of disability and that this association is reinforced if one experiences severe limitations or is a woman.

## 4. Results

### 4.1. Comparison of the Characteristics of the Populations with and without Disability

Some EISS-2012 health indicators show significant differences between individuals with and without disabilities in terms of perceptions of health, experiencing long-term or chronic illnesses, limitations to performing everyday activities, and most of the illnesses reported in the survey (see Table 3, Table 4, Table 5 and Table 6).

**Figure 2:**
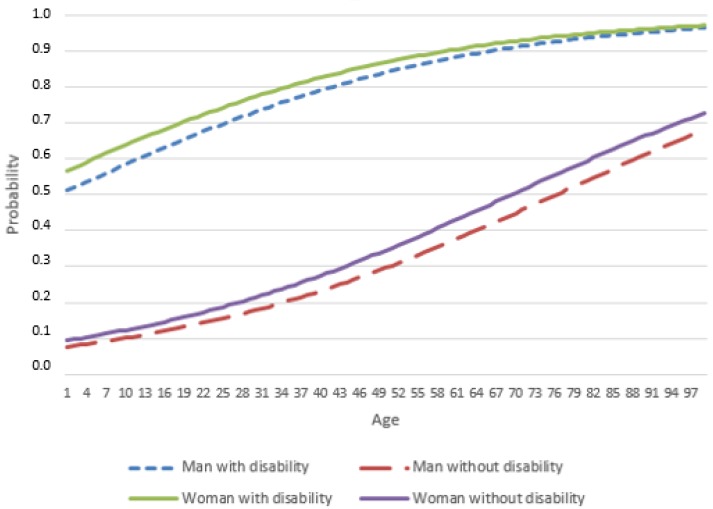
Probability of experiencing a chronic illness, by sex, age and having disability *. Source: Authors’ own from EISS-2012 data. (*) The regression model fits (Nagelkerke’s R2 = 0.327) satisfactorily, correctly predicting 73% of the sample cases as to whether they will or will not experience chronic health problems. The expression used to calculate the probability of experiencing a chronic illness is defined by: Π = [1/(1 + *e*^−[(−2.516 + (0.226·Sex) + (0.033·Age) + (2.525·Disability)]^].

Differences between PWD and Pw/oD with regard to certain health indicators include:

- Fifteen percent of PWD believe they have a good or very good health status compared to 80.5% of Pw/oD. On the other hand, 35.2% of PWD believe they have a bad or very bad state of health compared to 1.5% of Pw/oD.
- Eighty-nine percent of PWD experience some chronic or long-term health problem compared to 32% of Pw/oD.
- Twenty-four percent of PWD have experienced severe limitations in conducting everyday activities, due to health problems compared to 1% of Pw/oD.
- All groups of illnesses are more likely to be present in PWD than in Pw/oD (see Table 2). This is especially true of degenerative diseases (e.g., HIV, multiple sclerosis, Alzheimer’s disease and Parkinson’s disease), where the probability of a person with disability presenting one of these diseases is 94.7% with respect to a person without disability. For joint diseases, mental problems and cancer, these figures are 89.5%, 87.7%, and 87%, respectively.
- If we analyze only PWDs, we find that women have a higher incidence of articular diseases (women = 87.1%; men = 75.8%), allergies (28.1% vs. 19.9%), digestive disorders (37.6% vs. 30.8%), headaches and migraines (29.9% vs. 17%), anxiety (29% vs. 17.5%) and depression (29.2% vs. 16.6%), while men have a higher incidence of respiratory problems (men = 29.1%; women = 23.2%) and tumors (8.8% vs. 6.5%). On the other hand, no significant differences are found between the sexes when it comes to diabetes, heart disease, skin disease, epilepsy or degenerative diseases. A t-test for the mean of the cumulative sum of illnesses for men and women with disabilities shows that women with disabilities accumulate a greater number of diseases than men with disabilities (*t* = 10.198, *p* < 0.000).
- The OR in Table 4 decrease as age increases, i.e., there is a tendency for the prevalence of the various diseases among the population with and without disability to converge as age increases. This trend is observed in both men and women, but most of the OR for women are lower than those for men. This indicates that the differences between women with and without disabilities are smaller than those between men with and without disabilities (except for diabetes at all ages, mental problems, heart and circulatory problems, articular problems at younger ages, and digestive problems in middle age, where the difference between the probability of women with disability and the probability of women without disability experiencing these problems is greater than the difference for men).
- The probability of experiencing a long-term or chronic illness increases with age and disability (see Figure 2). Women with disability are more likely to experience a chronic disease than men with disability. The probabilities that women and men with a disability will experience a long-term or chronic illness converge as they get older. Women without disability are also more likely to experience a chronic illness than men without disability. However, unlike the convergence of the probabilities of men and women with disabilities, the probability that women without disability will experience a chronic illness increases compared to that of men without disability as they get older.

The EISS-2012 data we analyzed corroborate the findings in the literature on the association between health problems, one’s perception of these health problems, and whether one experiences some form of disability. However, they do not help us to determine whether the disability is prior to contracting a chronic illness or subsequent to it (though we may assume that, in many cases, chronicity and disability feed back on each other). Poor people have a greater incidence of articular diseases (52.5% of poor vs. 43% of non-poor), heart diseases (27% vs. 23.3%), digestive diseases (18.9% vs. 13.9%), chronic depression (9.2% vs. 5.7%) and chronic anxiety (10.8% vs. 6.7%). Diseases, such as allergies, tumors and epilepsy, show no statistically significant differences between the poor and the non-poor. Also, the poor experience a higher average number of diseases than the non-poor (*t* = 11.874, *p* < 0.000). The data also indicate statistically significant relationships with variables that in the literature frequently appear as determinants of risk of poverty [29].

Table 7 shows that in the extreme age groups (up to 30 years old and after 65) the probability of people with disability experiencing a situation of poverty is similar to that of people without disability, and that the probability of people with disability experiencing a situation of poverty is highest in the 30–65 age group. This same situation occurs when the control variables are experiencing chronic health problems and experiencing severe limitations. In no cases are there significant differences between men and women.

In the Table 7 and Table 8, in the OR analyzes the variables were adjusted for sex, therefore can there are interactions between some variables that do not disallow the contingency analysis. In the subsequent logistic regression analysis, the step-by-step method guarantees the best adjusted model.

The EISS-2012 data also corroborate the findings in the literature on the association between experiencing health problems, having some form of disability, and living in poverty. Not having a secure job is also associated with a higher risk of poverty, as is being a woman and completing only compulsory secondary education (or, more strongly, completing only primary studies or having no education at all).

At this juncture we are interested in discovering whether social exclusion is related to variables, such as being disabled, having a chronic illness, being severely limited in conducting activities, or living below the poverty line. In our analysis we include gender, educational level (i.e., compulsory or post-compulsory education), and whether the individual has a secure job. The OR resulting from the intersection between sociodemographic variables and health with social exclusion show the significance of these relationships. To calculate the ORs, we dichotomized the polytomous variables while always keeping as a reference a category in which the characteristic in question is provided absolutely, e.g., experiencing chronic health problems, having disability, or presenting severe limitations. For the variable *level of studies*, the dichotomy is between having completed a level of studies below or equal to those of compulsory education or having completed studies above the level of compulsory education. For the variable *employment situation*, the dichotomy is between having or not having a secure job.

Table 8 shows the OR corresponding to the variables in which the first category is the reference. We can see that there is a 92.6% probability that social exclusion is associated with having a disability as opposed to not having it and a 70.6% probability that it is associated with living in poverty as opposed to not living in poverty. Social exclusion also has important associations with having severe limitations (81.4%) and having chronic health problems (73.9%). The OR in this table show that the probability of social exclusion increases as age increases for those experiencing disability, chronic illness and severe limitation. However, the highest OR are observed for those experiencing disability, which is a stronger determinant of social exclusion than chronic illness or severe limitation. Also in this case, no differences are observed between men and women.

When we observe the average values of the net index of determinants of exclusion, we find that the mean comparison t tests for Pw/oD (=3.08) and PWD (=13.72) prove that there is a significant difference between the values for the two groups (t = 46.2; *p* < 0.000). Similarly, the mean for the group with a chronic illness ($\bar{x}=7.37$) is significantly different (*t* = 31.3; *p* < 0.000) from the mean for the group without a chronic illness ($\bar{x}=3.33$). To verify whether significant differences exist between the means of the groups that present limitations, we conducted one-factor ANOVA analysis. This showed that the three groups considered, i.e., (a) people with severe limitations, (b) people with moderate limitations, and (c) people without limitations, also had statistically significant means in the index ($\bar{x}=16.28$, $\bar{x}=7.74$, and $\bar{x}=3.23$, respectively).

We also found that for 42% of those with social exclusion (1794 out of 4271) the exclusion may be regarded as severe. The population experiencing severe exclusion represents 12.3% of the total sample. This group is largely made up of individuals with some form of disability (75% of those with severe exclusion), with severe or moderate limitations (74.2%), with long-term or chronic health problems (77, 3%), with only compulsory secondary education (44%), or only primary education (33.7%), in single-person homes (24.8%) or homes with couples with children (26.3%), or who are women (67.7%), or have an average age of 58.4 years.

### 4.2. Predictions of the Probabilities of Exclusion

To complete our analysis, we used a binary logistic regression model to determine the equation for calculating the probability of experiencing social exclusion. We did not include the variables *training* or *employment situation* in the model, because much of the sample remained outside the analysis, due to non-responses. The model is therefore based mainly on health variables (see Table 9).

The equation used to calculate the probability of being in a situation of exclusion is therefore:

$$\Pi =\frac{1}{1+e^{-[\left(-2.03+\left(0.172\cdot \mathrm{Sex}\right)+\left(0.832\cdot \mathrm{Poor}\right)+\left(0.157\cdot \text{Sev.lim}\right)+\left(0.78\cdot \mathrm{Non}-\text{sev.lim}\right)+\left(1.844\cdot \mathrm{Disab}\right)+\left(0.08\cdot \mathrm{Disea}\right)+\left(0.482\cdot \mathrm{MP}\right)+\left(0.032\cdot \mathrm{AD}\right)\right]}}$$

The regression coefficients indicate positive relationships with social exclusion. In our model, therefore, the risk of being excluded increases if one is a woman, has a disability with severe limitation, or is poor.

The probability of exclusion increases with age in individuals with disability, those who are poor, those with chronic health problems and those with severe limitations (see Table 10). However, the probability of exclusion hardly changes with age in individuals that do not experience these problems. In both men and women, the factors that have the greatest probabilities of producing social exclusion are experiencing severe limitations, followed by experiencing disability. In all cases, the chances of experiencing social exclusion are greater for women than for men.

The profiles of the men and women without disability lie beyond the influence of determinants of social exclusion, except for those who experience severe limitations. Again, therefore, this shows the importance that experiencing severe limitations has for carrying out everyday activities. Restrictions, due to severe limitations, mainly involve problems entering buildings and other areas (68% of people with severe limitations) and, to a lesser extent, not being able to enjoy leisure activities (17%), not going to cultural events (16%), and mobility restrictions (19%). For those without disabilities, therefore, two conditions must exist simultaneously for there to be a risk of social exclusion: Being poor and experiencing a severe limitation.

On the other hand, all profiles of individuals with disabilities present probabilities above 0.5 of accumulating determinants of social exclusion (see Table 11). For those with disabilities, severe or moderate limitations and being or not being poor can increase the intensity of social exclusion, which means that the probability of experiencing social exclusion is greater. Individuals most likely to experience exclusion are poor women with disability and severe limitation, and whose probability of accumulating the maximum number of determinants of social exclusion is 0.92. The exclusion probability of a man with disability, but who is not poor and has no limitation is 0.55. Exclusion probabilities decrease when the limitations are less severe, and the level of poverty is lower. However, the reduction in probability is not sufficient for this group with disabilities to remain exempt from exclusion.

Women with and without disabilities are always more likely than men to experience exclusion.

## 5. Discussion

Social exclusion, as a multidimensional concept, is the result of a combination of social vulnerability situations, including factors such as disability, chronic illness, severe limitation in performing certain activities, and poverty. Poverty and social exclusion are concepts that can only be explained by the interaction of health-related situations, including whether or not the individual experiences disability. We have proposed a social exclusion construct that includes the dimensions of health (the chronicity of illness), functioning (limitations in carrying out daily activities), social involvement (leisure and social relations) and poverty from a multidimensional perspective.

Our analysis reveals the interdependence between health status, disability and risks of poverty on the one hand and social exclusion on the other. Compared to Pw/oD, PWD have more negative perceptions of their state of health (35.2% of PWD consider their health to be bad or very bad compared to 1.5% of Pw/oD), higher levels of chronic or long-term illness (89.3% vs. 32%), greater risk of poverty (33.4% vs. 23.7%), and higher scores on the determinants of social exclusion index (74.6% vs. 19%). We have also shown that PWD are more likely to accumulate determinants that involve some level of social exclusion: PWD accumulate an average of 13.72 on the Determinants of Social Exclusion index compared to an average of 3.08 for Pw/oD.

Although disability is not in itself a condition that leads to poverty or social exclusion, the data reveal statistical relationships between having a disability and being at greater risk of social exclusion. These relationships are accompanied by other factors that act as contributory variables, such as having severe limitations or experiencing chronic illness. Being a woman is another factor that increases the probability of living in poverty or being socially excluded (the former being more likely than the latter). We believe that detailed studies should be conducted to ascertain how gender influences the exclusion processes and the mechanisms that cause it.

Having analyzed the data, we believe that the distinction between congenital and acquired disability is not as relevant as it initially appeared to be, since more people are experiencing disability in adulthood. This is especially true of those over 65.

Our analysis has confirmed that the prevalence of chronic illness increases with age. Some chronic illnesses affect women more than men (e.g., articular and digestive diseases, migraines, depression and anxiety), while others affect men more than women (respiratory diseases and tumors). Also, chronicity is more present among those who experience disability.

Our analysis of the OR shows that the probability of social exclusion increases with age for those with disability, chronic illness and severe limitation. It is interesting to observe, however, that disability is a stronger determinant of social exclusion than chronic illness or severe limitation.

The logistic regression equation has also shown that women with disabilities are the individuals most likely to experience situations of social exclusion. Having or not having a disability is decisive when it comes to increasing the risk of social exclusion. Severe limitation correlates with disability, so we find many more situations of severe limitations among men and women with disabilities than among men and women without.

The highest probabilities of experiencing social exclusion are found among poor women with disabilities and severe limitations in conducting their activities (0.9237). The corresponding probability for men is 0.9007. At the opposite extreme, men without disabilities and without limitations and who are not poor have a probability of experiencing social exclusion of 0.1293, while the probability for women in the same situation is 0.1653. The probability of women experiencing social exclusion is always greater than it is for men.

## 6. Conclusions

The data obtained from our analysis allow us to accept our working hypothesis: The probability of experiencing social exclusion is higher for individuals with disability, especially if in addition to their disability they are poor and have severe limitations. This is applicable to both men and women, though the probability of women experiencing social exclusion is always higher in all situations.

Our analysis highlights two groups to which special attention should be paid when public administrations formulate policies aimed at reducing the determinants that affect them. One is the group of women living in poverty with disabilities, severe limitations and chronic illness (*n* = 438). This group represents 3% of the total population and 5.5% of all women. It also represents 13.1% of the total number of people in poverty and 23% of women in poverty, as well as 16.3% of the total population with some form of disability and 25.3% of all women with disability. The average age of these women is 63, 38.4% of them live alone, their probability of social exclusion is 0.863, and their average number of accumulated illnesses (see Table 1) is 6.02. The second group to which special attention should be paid is that of men (*n* = 211) with a slightly lower average age (58.5 years), a probability of social exclusion of 0.828, and an average number of accumulated illnesses of 5.14. These men represent 3.1% of the total number of men, 14.9% of the total number of poor men and 21.9% of the total number of men with disabilities.

The analysis we present in this paper could help public administrations and entities working in the third sector to identify the most vulnerable groups in society. Measures aimed at combating poverty or social exclusion should not be limited to financial allowances, since comprehensive action should go beyond material provisions. This presents interesting challenges for public actions (institutional or otherwise) that are intended to tackle situations which are becoming ever more frequent in our society, due to factors such as an ageing population, longer life expectancy, unstable employment, and the breakdown in social bonds.

Future lines of research should focus on more specific analyses of the problems surrounding disability. This requires the availability of disaggregated financial data on individuals with disabilities and their households so that income from transfers can be separated from income from other sources. This is the only way to obtain more precise estimates of the impact of monetary transfers on reductions in poverty for people with disabilities.

Another interesting line of study would be to compare the problems associated with disabilities, due to accidents with those associated with other forms of disability. Unfortunately, when analyzing people with disabilities we are rarely able to differentiate in this way. Some of those classified as disabled are really dependents, generally of legal age, who present common problems or other problems different from those of individuals recognized as being disabled since childhood, for example. Although, ultimately, there are concomitants between these two large populations, their specific situations should be analyzed to obtain better and more detailed knowledge of their profiles. This would enable public administrations and other entities to deal more effectively with problems arising in the field of social care.

Finally, we should point out the limitations of conducting surveys at the household level to ascertain the vulnerability of certain people. A special case is that of homelessness, a phenomenon on which the most aggressive determinants of vulnerability converge, i.e., the lack of a home, institutional abandonment, the loss of social bonds, financial precariousness, exposure to disease and frequent chronic disabling ailments, many of which are mental-health related. In this regard, the 2012 Homeless Persons Survey (*Encuesta sobre las Personas sin Hogar*) published by the INE [30] revealed that 30.7% of the homeless people interviewed experienced a chronic illness and that 16.6% recognized experiencing mental disorders. These illnesses were more frequent in women (22.3%) than in men (15.0%). It was also reported that 15.2% had some form of recognized disability.

## Figures and Tables

**Table 1:** Distribution of the sample by age and gender.

	**Total Sample**		**With Disability**		**Without Disability**	
			**Men (*n* = 964)**	**Women (*n* = 1732)**	**Men (*n* = 5695)**	**Women (*n* = 6223)**
**Age (years)**	**Frequency (*n*)**	**Percent (%)**	**Frequency (*n*)**	**Frequency (*n*)**	**Frequency (*n*)**	**Frequency (*n*)**
15–30	1946	13.3	46	59	895	946
31–47	4896	33.5	179	272	2199	2246
48–65	4332	29.6	343	500	1643	1846
66+	3440	23.5	396	901	958	1185
Total	14,614	100	964	1732	5695	6223
	**Total Sample**		**With Disability**		**Without Disability**	
			**Men (*n* = 964)**	**Women (*n* = 1732)**	**Men (*n* = 5695)**	**Women (*n* = 6223)**
**Age (years)**	**Frequency (*n*)**	**Percent (%)**	**Percent (%)**	**Percent (%)**	**Percent (%)**	**Percent (%)**
15–30	1946	13.3	4.8	3.4	15.7	15.2
31–47	4896	33.5	18.6	15.7	38.6	36.1
48–65	4332	29.6	35.6	28.9	28.8	29.7
66+	3440	23.5	41.1	52.0	16.8	19.0
Total	14,614	100	100	100	100	100

Source: Authors’ own from Social Integration and Health Survey (EISS)-2012 data.

**Table 2:** Weighting factors for each dimension.

Dimensions Analyzed	Correlation with Component 1	Weighting Factor
Mobility	0.7584	0.25985
Accessibility	0.6560	0.22477
Contact and social support	0.5682	0.19468
Employment	0.3214	0.11012
Education	0.2541	0.08706
Leisure	0.2354	0.08066
Internet access	0.1251	0.04286

Source: Authors’ own from EISS-2012 data.

**Table 3:** Chronic illnesses or ailments of individuals with and without disabilities.

Chronic Illnesses or Ailments	Pw/oD	PWD	OR	Transformed OR (*)
1. Problems in the arms and hands (including arthritis and rheumatism)	13.9%	54.0%	7.2712	87.9%
2. Problems in the legs and feet (including arthritis and rheumatism)	17.2%	65.8%	9.2662	90.3%
3. Problems in the back and neck (including arthritis and rheumatism)	25.5%	64.7%	5.3492	84.3%
4. Heart and circulation problems, such as high blood pressure (including strokes with long-term consequences)	17.3%	52.7%	5.3288	84.2%
5. Allergies, such as rhinitis, eye inflammation, dermatitis, food allergies (including allergic asthma)	18.5%	25.1%	1.4816	59.7%
6. Respiratory problems (including asthma and chronic bronchitis; (excluding allergic reactions, such as allergic asthma)	5.9%	25.3%	5.4046	84.4%
7. Stomach, liver, kidney and digestive problems (excluding allergic reactions)	10.5%	35.2%	4.6402	82.3%
8. Skin diseases (including general disfiguration (excluding allergic reactions, such as dermatitis)	4.0%	12.0%	3.2791	76.6%
9. Diabetes	5.1%	20.0%	4.6618	82.3%
10. Cancer (malignant tumors; including leukemia and lymphoma)	1.2%	7.3%	6.7169	87.0%
11. Epilepsy (including attacks)	0.3%	2.3%	7.2879	87.9%
12. Strong headaches (such as migraines)	9.9%	25.3%	3.0815	75.5%
13. Chronic anxiety	3.8%	24.9%	8.5169	89.5%
14. Chronic depression	2.5%	24.7%	12.705	92.7%
15. Learning difficulties (reading, writing, mathematics)	2.1%	20.3%	12.049	92.3%
16. Other mental, nervous or emotional problems	3.5%	22.9%	8.1046	89.0%
17. Other degenerative diseases (such as HIV, multiple sclerosis, Alzheimer’s disease and Parkinson’s disease)	0.7%	11.1%	18.073	94.8%
18. Other long-term illnesses or health conditions	6.5%	23.0%	4.3119	81.2%

(*) All prevalences present significant statistical associations with *p* < 0.000. Source: Authors’ own from EISS-2012 data. PWD, individuals who reported some form of disability; OR, Odds Ratios.

**Table 4:** Prevalence of health problems by gender, age, and whether the individual experiences disability.

Chronic Illnesses or Ailments	Men (*n* = 6659)								Women (*n* = 7955)							
	Pw/oD (*n* = 5695)				PWD (*n* = 964)				Pw/oD (*n* = 6223)				PWD (*n* = 1732)			
	15–30	31–47	48–65	66+	15–30	31–47	48–65	66+	15–30	31–47	48–65	66+	15–30	31–47	48–65	66+
1. Problems in the arms and hands (including arthritis and rheumatism)	3.1%	4.7%	11.3%	17.3%	21.7%	25.1%	41.4%	41.4%	2.6%	9.0%	26.8%	38.3%	22.0%	37.5%	69.0%	70.5%
2. Problems in the legs and feet (including arthritis and rheumatism)	5.4%	8.2%	15.0%	27.7%	43.5%	40.2%	57.4%	66.7%	5.0%	10.2%	27.2%	45.0%	37.3%	46.0%	71.2%	79.8%
3. Problems in the back and neck (including arthritis and rheumatism)	9.4%	15.9%	24.3%	24.6%	30.4%	50.3%	56.9%	51.8%	15.9%	26.0%	39.8%	42.8%	40.7%	59.6%	80.4%	72.5%
4. Heart and circulation problems, such as high blood pressure (including strokes with long-term consequences)	2.1%	6.0%	24.8%	41.2%	13.0%	22.9%	51.6%	65.2%	2.2%	6.1%	21.7%	46.7%	16.9%	26.5%	46.4%	69.5%
5. Allergies, such as rhinitis, eye inflammation, dermatitis, food allergies (including allergic asthma)	21.8%	19.5%	13.5%	10.6%	34.8%	22.9%	21.3%	15.7%	20.2%	22.8%	19.7%	16.0%	37.3%	34.6%	30.0%	24.4%
6. Respiratory problems (including asthma and chronic bronchitis; (excluding allergic reactions, such as allergic asthma)	5.3%	4.1%	6.4%	12.8%	23.9%	17.9%	25.1%	38.4%	4.3%	4.4%	6.1%	7.3%	18.6%	16.5%	23.2%	25.5%
7. Stomach, liver, kidney and digestive problems (excluding allergic reactions)	3.6%	8.6%	11.6%	13.7%	15.2%	28.5%	31.5%	33.1%	7.2%	8.6%	12.5%	18.1%	22.0%	34.2%	42.6%	37.0%
8. Skin diseases (including general disfiguration); (excluding allergic reactions, such as dermatitis)	2.2%	3.3%	3.0%	5.5%	2.2%	14.0%	11.1%	15.7%	4.1%	4.5%	4.7%	4.7%	5.1%	13.2%	11.4%	11.2%
9. Diabetes	1.0%	1.7%	8.6%	16.4%	4.3%	4.5%	24.8%	27.8%	0.7%	1.2%	5.0%	11.2%	5.1%	8.1%	14.8%	26.0%
10. Cancer (malignant tumors; including leukemia and lymphoma)	0.2%	0.1%	1.5%	3.8%		2.8%	9.0%	12.4%		0.4%	1.7%	2.6%	1.7%	7.0%	7.6%	6.1%
11. Epilepsy (including attacks)	0.1%	0.1%	0.3%		4.3%	5.0%	2.3%	2.0%	0.1%	0.8%	0.2%	0.7%	3.4%	2.9%	2.8%	1.3%
12. Strong headaches (such as migraines)	7.5%	5.5%	5.6%	4.0%	15.2%	24.0%	14.3%	16.4%	12.6%	16.8%	14.1%	8.9%	37.3%	39.7%	38.4%	21.8%
13. Chronic anxiety	1.2%	1.9%	2.6%	2.3%	8.7%	19.6%	21.6%	14.1%	2.4%	4.0%	7.9%	6.8%	22.0%	29.4%	38.8%	24.0%
14. Chronic depression	0.6%	0.7%	1.6%	2.6%	10.9%	16.2%	18.1%	16.2%	0.7%	1.8%	5.1%	7.3%	18.6%	22.8%	34.8%	28.7%
15. Learning difficulties (reading, writing, mathematics)	1.9%	0.8%	1.6%	4.6%	19.6%	15.6%	12.8%	23.7%	1.2%	0.9%	1.6%	6.8%	20.3%	10.3%	15.8%	28.0%
16. Other mental, nervous or emotional problems	1.5%	2.4%	2.9%	3.1%	17.4%	22.9%	20.4%	23.2%	3.1%	3.3%	5.1%	6.8%	25.4%	23.5%	23.6%	23.2%
17. Other degenerative diseases (such as HIV, multiple sclerosis, Alzheimer’s disease and Parkinson’s disease)	0.3%	0.3%	0.5%	1.5%	2.2%	11.2%	7.3%	12.9%	0.1%	0.6%	0.9%	1.6%		7.7%	10.2%	14.5%
18. Other long-term illnesses or health conditions	1.9%	3.5%	6.9%	7.4%	26.1%	19.6%	20.4%	23.2%	3.7%	7.3%	9.5%	10.0%	22.0%	27.9%	26.0%	21.3%
All prevalences present significant statistical associations with *p* < 0.000																

Source: Authors’ own from EISS-2012 data.

**Table 5:** OR corresponding to experiencing illness and having disability.

Chronic Illnesses or Ailments	OR	OR Confidence Interval	Probability That the Illness Is Associated with PWD	Haenszel-Mantel Chi-Square
Articular disease	8.521	[7.74; 9.38]	89.5%	43.83 **
Heart and circulation problems	5.329	[4.89; 5.79]	84.2%	38.97 **
Allergic problems	1.481	[1.34; 1.63]	59.7%	7.85 **
Respiratory diseases	5.405	[4.86; 6.01]	84.4%	31.07 **
Digestive diseases	4.64	[4.23; 5.09]	82.3%	32.42 **
Skin diseases	3.279	[2.84; 3.77]	76.6%	16.49 **
Diabetes	4.662	[4.15; 5.23]	82.3%	25.98 **
Cancer	6.717	[5.53; 8.15]	87.0%	19.29 **
Mental problems	7.142	[6.56; 7.76]	87.7%	46.01 **
Degenerative diseases	18.073	[15.02; 21.7]	94.8%	30.68 **

** Test significance *p* < 0.000. Source: Authors’ own from EISS-2012 data.

**Table 6:** OR corresponding to experiencing illness and having disability, by sex and age.

Chronic Illnesses or Ailments	Men				Women			
	15–30	31–47	48–65	66+	15–30	31–47	48–65	66+
Articular disease	8.757	8.776	6.009	4.618	5.958	5.284	8.404	5.242
Heart and circulation problems	6.916	4.690	3.227	2.665	8.989	5.585	3.119	2.601
Allergic problems	1.914	1.229	1.739	1.557	2.350	1.784	1.751	1.692
Respiratory diseases	5.670	5.043	4.901	4.229	0.531	4.345	4.633	4.380
Digestive diseases	4.840	4.237	3.494	3.121	3.649	5.495	5.214	2.66
Skin diseases	*	4.796	4.053	3.170	1.245	3.274	2.544	2.545
Diabetes	4.475	2.660	3.482	1.962	7.186	7.232	3.312	2.775
Cancer	0.971	21.034	6.430	3.617	*	16.792	4.663	2.4201
Mental problems	5.750	9.406	6.804	7.787	11.868	6.694	6.831	4.911
Degenerative diseases	6.607	39.389	14.273	9.967	*	14.371	12.991	10.441

Source: Authors’ own from EISS-2012 data. (*) No data available.

**Table 7:** OR corresponding to living in poverty and certain variables.

Variables Analyzed	OR	OR Confidence Interval (95%)	Probability That Poverty Is Associated with These Situations	Haenszel-Mantel Chi-Square
Employment status: Non-secure employment	1.79	[1.54; 2.08]	64.2%	7.74 *
Sex: Female	1.22	[1.12; 1.32]	54.8%	4.82 *
Education: Compulsory-level only	2.70	[2.46; 2.95]	72.9%	21.7 **
Disability: YES	1.61	[1.46; 1.77]	61.6%	9.80 *
Disability: Age: 15–30	0.80	[0.48; 1.31]	44.5%	0.88
Disability: Age: 31–47	2.58	[2.11; 3.16]	72.1%	9.17 **
Disability: Age: 48–65	1.89	[1.60; 2.23]	65.4%	7.49 *
Disability: Age: +65	1.19	[1.01; 1.41]	54.5%	2.16
Chronic health problems: YES	1.33	[1.23; 1.44]	57.0%	7.03 *
Chronic health problems: Age: 15–30	0.93	[0.70; 1.24]	48.4%	0.44
Chronic health problems: Age: 31–47	1.51	[1.30; 1.74]	60.1%	5.49 *
Chronic health problems: Age: 48–65	1.51	[1.30; 1.74]	60.1%	5.61 *
Chronic health problems: Age: +65	1.11	[0.93; 1.32]	52.7%	1.23
Severe limitations: YES	1.61	[1.37; 1.89]	61.6%	5.79 *
Severe limitations: Age: 15–30	0.55	[0.18; 1.61]	35.5%	1.08
Severe limitations: Age: 31–47	3.00	[2.02; 4.45]	75.0%	5.48 *
Severe limitations: Age: 48–65	1.78	[1.33; 2.38]	64.0%	3.91 *
Severe limitations: Age: +65	1.29	[1.01; 1.63]	56.3%	2.11

* *p* < 0.05; ** *p* < 0.01; Source: Authors’ own from EISS-2012 data.

**Table 8:** OR corresponding to social exclusion and certain factors.

Variables Analyzed	OR	OR Confidence Interval (95%)	Probability That Social Exclusion Is Associated with These Situations	Haenszel-Mantel Chi-Square
Employment status: non-secure employment	1.41	[1.23; 1.60]	58.47%	5.07 *
Sex: Female	1.58	[1.46; 1.70]	61.25%	12.34 **
Education: Compulsory-level only	2.23	[2.05; 2.41]	69.02%	19.95 **
Disability: YES	12.57	[11.5; 13.7]	92.63%	57.39 **
Disability: Age: 15–30	9.12	[6.28; 13.2]	90.1%	11.6 **
Disability: Age: 31–47	13.06	[10.7; 15.8]	92.8%	25.5 **
Disability: Age: 48–65	13.75	[11.7; 16.1]	93.2%	32.3 **
Disability: Age: +65	30.1	[25.4; 35.7]	96.7%	38.9 **
Chronic health problems: YES	2.83	[2.63; 3.04]	73.89%	28.13 **
Chronic health problems: Age: 15–30	1.82	[1.41; 2.33]	65.5%	4.72 *
Chronic health problems: Age: 31–47	2.58	[2.27; 2.95]	72.1%	14.23 **
Chronic health problems: Age: 48–65	3.01	[2.64; 3.45]	75.1%	16.15 **
Chronic health problems: Age: +65	5.01	[4.17; 6.03]	83.4%	17.22 **
Severe limitations: YES	4.40	[4.08; 4.74]	81.48%	39.13 **
Severe limitations: Age: 15–30	4.82	[2.30; 10.0]	82.8%	4.2 *
Severe limitations: Age: 31–47	8.53	[5.82; 12.5]	89.5%	10.96 **
Severe limitations: Age: 48–65	8.16	[6.22; 10.7]	89.1%	15.17 **
Severe limitations: Age: +65	13.06	[10.9; 16.9]	93.1%	23.21 **

* *p* < 0.05; ** *p* < 0.01; Source: Authors’ own from EISS-2012 data.

**Table 9:** Equation variables.

Variables in the Equation	B	S.E.	Wald	df	Sig.	Exp(B)	95% CI for EXP(B)	
							Lower	Upper
Sex (Female)	0.172	0.046	13.835	1	0.0000	1.188	1.085	1.301
Poverty (Yes)	0.832	0.049	293.433	1	0.0000	2.298	2.089	2.527
Limitation (Non-several)	0.78	0.121	41.493	1	0.0000	2.181	1.72	2.765
Limitation (Several)	0.157	0.059	6.976	1	0.008	1.17	1.041	1.315
Disability (Yes)	1.844	0.068	731.918	1	0.0000	6.32	5.53	7.223
Sum of diseases	0.08	0.017	23.049	1	0.0000	1.083	1.048	1.119
Mental problems (Yes)	0.482	0.061	63.115	1	0.0000	1.62	1.438	1.824
Articular diseases (Yes)	0.032	0.06	0.293	1	0.588	1.033	0.919	1.161
Constant	−2.03	0.043	2237.294	1	0.0000	0.131		
Variable(s) entered on step 1: Sex, Poverty, Limitation, Disability, Sum of diseases, Mental problems, Joint problems.								

R^2^ de Nagelkerke = 0.323; Predicted overall percentage = 79.3%. Source: Authors’ own from EISS-2012 data.

**Table 10:** Probabilities of social exclusion according to different profiles.

Different Profiles	Male					Female				
	15–30	31–47	48–65	66+	Total	15–30	31–47	48–65	66+	Total
With disability	0.6736	0.7145	0.7093	0.7032	0.7026	0.7381	0.7621	0.7705	0.7772	0.7731
Without disability	0.1737	0.1634	0.1750	0.1743	0.1695	0.2283	0.2097	0.2201	0.2346	0.2185
Poor	0.2893	0.3514	0.4085	0.4498	0.3791	0.3668	0.4304	0.5009	0.5904	0.4893
Non-poor	0.1646	0.1621	0.2191	0.2859	0.2052	0.2084	0.2191	0.2744	0.2915	0.2484
Chronic health problems	0.2935	0.3114	0.3617	0.3953	0.3671	0.3568	0.3818	0.4370	0.5461	0.4768
Non-chronic health problems	0.1807	0.1676	0.1772	0.2077	0.1716	0.2272	0.2150	0.2261	0.2913	0.2183
Mental Problems	0.2758	0.3478	0.4454	0.5277	0.5052	0.35505	0.3744	0.4778	0.6099	0.5504
Non-mental Problems	0.1889	0.1842	0.2258	0.2621	0.1968	0.2293	0.2316	0.2668	0.2881	0.2540
Severe limitations	0.6626	0.6744	0.7535	0.7715	0.7115	0.7683	0.7899	0.7854	0.8361	0.7949
Non-severe limitations	0.1909	0.1945	0.2372	0.2841	0.2267	0.2491	0.2583	0.3118	0.3532	0.2931

Source: Authors’ own from EISS-2012 data.

**Table 11:** Probabilities of social exclusion according to different profiles.

Different Profiles	Female		Male	
	With Disability	Without Disability	With Disability	Without Disability
Poor with severe limitation	0.9293	0.5690	0.8976	0.5135
Poor with moderate limitation	0.8469	0.4117	0.8058	0.3369
Poor with no limitation	0.8012	0.3179	0.7419	0.2619
Non-poor with severe limitation	0.8409	0.3753	0.7907	0.2931
Non-poor with moderate limitation	0.7009	0.2254	0.6319	0.1726
Non-poor with no limitation	0.6340	0.1638	0.5522	0.1306

Source: Authors’ own from EISS-2012 data.
